# Extending the Eisenbarth Model: Stage 0 as a Provisional Framework for Early Risk Stratification and Prevention in Type 1 Diabetes

**DOI:** 10.1155/jdr/9970365

**Published:** 2026-06-26

**Authors:** Maya Puhachova, Huda K. Klair, Balamurali Hariharan, Iffat Imtiaz, Juan José Zambrano Valenzuela, Essra Hassan Adam Alradi, Elizabeth Gbobbo, Faiqa Zahoor, Hassan Shah, Viraj Kale, Niketa Narasimhan, Fadi Ali Jamaleddin Ahmad

**Affiliations:** ^1^ Faculty of Medicine and Surgery, Catholic University of the Sacred Heart, Rome, Italy, unicatt.it; ^2^ Department of Medicine, FMH College of Medicine and Dentistry, Lahore, Punjab, Pakistan; ^3^ Department of Medicine, PSG Institute of Medical Sciences and Research, Coimbatore, Tamil Nadu, India, psgimsr.ac.in; ^4^ Department of Medicine, University of Dhaka, Dhaka, Bangladesh, du.ac.bd; ^5^ Faculty of Medicine, National University of Colombia, Bogota, Colombia, unal.edu.co; ^6^ Department of Medicine, Dalian Medical University, Dalian, Liaoning, China, dlmedu.edu.cn; ^7^ Department of Medicine, All Saints University School of Medicine, Arnos Vale, Saint Vincent and the Grenadines, allsaintsuniversity.org; ^8^ Department of Medicine, Samaritan Medical Center, Watertown, New York, USA; ^9^ Department of Medicine, Shahjalal University of Science and Technology, Sylhet, Bangladesh, sust.edu; ^10^ Department of Medicine, Tver State Medical University, Tver, Russia; ^11^ Department of Biology, Miami University, Oxford, Ohio, USA, miami.edu; ^12^ Department of Medicine, American University of the Caribbean School of Medicine, Cupecoy, Saint Martin, aucmed.edu; ^13^ School of Medicine, University of New Mexico, Albuquerque, New Mexico, USA, unm.edu

**Keywords:** autoimmunity, biomarkers, primary prevention, risk stratification, Type 1 diabetes mellitus

## Abstract

**Background:**

Type 1 diabetes (T1D) is an autoimmune disease characterized primarily by T cell‐mediated pancreatic *β*‐cell destruction, with islet autoantibodies serving as important biomarkers of autoimmune activity and risk progression. Early detection of immune imbalances before seroconversion may help identify individuals at increased risk before established autoimmunity develops. In this review, the proposed “Stage 0” construct is framed as a hypothesis‐driven, preautoimmune research construct rather than an established clinical stage.

**Objective:**

This narrative review evaluates the proposed Stage 0 construct as a hypothesis‐driven, preautoimmune conceptual framework for T1D, summarizes genetic, environmental, metabolic, and immunological factors that may precede islet autoantibody seroconversion, and outlines research priorities for risk stratification and prevention.

**Methods:**

This review searched PubMed and Google Scholar using MeSH and free‐text terms to identify studies on early T1D pathogenesis, genetics, immunity, omics, metabolism, biomarkers, screening, and prevention. English‐language human studies, mechanistic studies, reviews, and selected animal studies were included when relevant to early T1D biology. The SANRA framework was used to assess methodological quality.

**Key Content and Findings:**

This review discusses Stage 0 as a proposed preautoimmune phase and evaluates factors that may affect T1D progression, including early signs of inflammation, metabolic changes, gut dysbiosis, and *β*‐cell stress. Polygenic and HLA‐based risk scores may improve disease prediction, but their performance differs across ancestries and requires population‐specific validation. The evidence remains strongest for genetic risk and islet autoantibody status, whereas many preautoantibody biomarkers remain exploratory and require replication. Prevention strategies are reviewed across immune‐modulating, antigen‐specific, metabolic, microbiome‐oriented, and screening‐linked pathways.

**Conclusion:**

Existing evidence supports additional research into preautoimmune biological alterations prior to the emergence of autoantibodies; however, Stage 0 should not be recognized as a clinical stage at this time. Standard biomarkers, ancestry‐inclusive risk models, and prospective validation are essential before Stage 0 screening is considered for routine practice. Future research should determine whether this provisional framework can be translated into ethical, evidence‐based screening and prevention pathways.

## 1. Introduction

**Figure 1:**
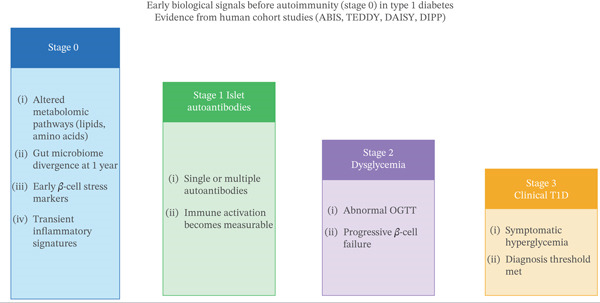
Key differences in the stages of Type 1 diabetes development [5, 6]. Abbreviations: ABIS, all babies in Southeast Sweden study; TEDDY, The Environmental Determinants of Diabetes in the Young; DAISY, Diabetes Autoimmunity Study in the Young; DIPP, Type 1 Diabetes Prediction and Prevention Study; OGTT, Oral Glucose Tolerance Test.

Type 1 diabetes (T1D) is an autoimmune condition characterized by progressive, predominantly T cell‐mediated beta (*β*)‐cell loss that results in total insulin dependence [1]. According to Eisenbarth′s model, T1D progresses from environmental exposures and genetic risk to autoimmune *β*‐cell loss, with clinical symptoms often emerging only after substantial loss of *β*‐cell functional reserve [2]. Early identification of at‐risk individuals has been enabled by advances in autoantibody detection, insulin autoantibodies (IAA), glutamic acid decarboxylase 65 (GAD65), insulinoma‐associated protein 2 (IA‐2), and zinc transporter 8 (ZnT8). Research indicates that children with two or more islet autoantibodies have a high long‐term risk of developing T1D [3]. The 2015 update from the American Diabetes Association (ADA), Endocrine Society (ES), and Juvenile Diabetes Research Foundation (JDRF) outlined three disease phases based on these findings [4]: Stage 1 involves multiple autoantibodies with normal glucose levels, Stage 2 includes autoantibodies with dysglycemia, and Stage 3 represents clinical diabetes; these stages are illustrated in Figure 1. The U.S. Food and Drug Administration (FDA) approved teplizumab in 2022 as the first treatment to delay the onset of clinical T1D, reflecting the clinical value of presymptomatic staging [7].

The current T1D staging approach formally begins at autoantibody seroconversion and therefore does not classify the interval between genetic susceptibility and the earliest immune activation. This gap is important because gene‐environment interactions may begin early in life, before autoantibody formation, and may contribute to disease initiation in genetically susceptible individuals [8]. Human leukocyte antigen (HLA) Class II alleles (DR3‐DQ2 and DR4‐DQ8) confer the highest risk, but more than 60 loci are linked to T1D; genetics alone cannot fully explain disease development. Monozygotic twin studies show only 30%–50% concordance, highlighting the importance of environmental and epigenetic factors during the putative preautoimmune stage [9, 10].

The proposed Stage 0 framework refers to a putative preautoimmune period in which genetic risk, normoglycemia, and candidate biological disturbances may precede islet autoantibody seroconversion [8, 11–13]. Longitudinal data suggest that immune, metabolic, and epigenetic alterations can appear months to years before seroconversion, but these findings do not yet define a clinical disease stage. Preseroconversion T cell autoreactivity, elevated inflammatory cytokine levels, altered dendritic cell function, and increased interferon‐related activity represent hypothesized transitional biology that may precede the emergence of autoantibodies [11]. Enteroviruses, cow′s milk proteins, early gluten introduction, vitamin D deficiency, gut dysbiosis, cesarean‐section birth, and antibiotic exposure are among the early environmental factors associated with risk in cohorts such as The Environmental Determinants of Diabetes in the Young (TEDDY) study [14]. Metabolic alterations may also precede antibody development, including changes in bile acids, oxidative stress indicators, and lipid and amino acid metabolism [12]. These findings support a research framework for risk enrichment rather than a validated diagnostic category [13].

Considering a proposed Stage 0 framework may help address gaps in current staging and shift prevention research from delaying established autoimmunity toward preventing or postponing its initiation [1, 15, 16]. However, there is no standardized framework for identifying or intervening in individuals before the development of autoantibodies. In this review, Stage 0 denotes a proposed preautoimmune phase in genetically predisposed individuals who remain islet autoantibody‐negative and normoglycemic. The terms “preautoimmune” and “preseroconversion” refer to the same putative interval; “preclinical T1D” is reserved for the confirmed autoantibody‐positive stages 1 and 2. The primary research question is, “What genetic, immune, metabolic, microbial, and environmental markers are associated with biological risk prior to the onset of islet autoantibodies, and how could these markers inform prevention research?” The aim is to consolidate emerging evidence that may support a Stage 0 framework and to clarify translational implications for screening, trial design, and public health, while recognizing uncertainty and limitations.

## 2. Methods

This review follows a narrative synthesis approach rather than a systematic review framework. A literature search was performed from October 1, 2025, to May 2026, encompassing English‐language peer‐reviewed articles, significant consensus statements, pertinent narrative and systematic reviews, and selected animal/mechanistic studies that elucidated biological plausibility, based on the available literature due to the scarcity of sources without specified target years for eligible papers. For this purpose, a comprehensive literature search was conducted in PubMed and Google Scholar using a specific search strategy that combined controlled vocabulary (MeSH) terms related to T1D, early or preclinical disease phases, HLA, polygenic risk, immunity, beta‐cell stress, microbes, screening, prediction, and prevention. Table 1 summarizes the databases, search strategy, screening process, inclusion rationale, quality assessment, and ethical considerations used in this review.

**Table 1:** Literature search strategy and review methodology.

Component	Description
Study design	Narrative review synthesizing existing literature on the conceptualization of Stage 0 in the progression of Type 1 diabetes (T1D) mellitus within the framework of the Eisenbarth model.
Databases searched	PubMed and Google Scholar.
Timeframe searched	October 1, 2025‐May 10, 2026.
Search strategy (PubMed)	A comprehensive search combining MeSH terms and free‐text terms related to early disease phases, pathophysiology, and omics technologies. The PubMed query was (“Diabetes Mellitus, Type 1” [MeSH] or “Type 1 Diabetes” [tiab] or T1D [tiab]) and (“preclinical” [tiab] or “early” [tiab] or “prodromal” [tiab] or “subclinical” [tiab] or “disease initiation” [tiab] or “pathogenesis” [tiab]) and (“HLA” [tiab] or “genetic” [tiab] or “polygenic” [tiab] or “immune” [tiab] or “autoimmunity” [tiab] or “cytokine” [tiab] or “T cell” [tiab] or “inflammation” [tiab]) and (“beta cell” [tiab] or “beta‐cell” [tiab] or “insulin secretion” [tiab] or “metabolic” [tiab] or “oxidative stress” [tiab]) and (“biomarker” [tiab] or “omics” [tiab] or “metabolomics” [tiab] or “proteomics” [tiab] or “transcriptomics” [tiab] or “screening” [tiab] or “prediction” [tiab]).
Search strategy (Google Scholar)	Targeted searches included “Type 1 diabetes risk genetic score,” “early nutrition in Stage 0 diabetes,” “Stage 0 Type 1 diabetes biomarkers,” “multi‐ancestry HLA Type 1 diabetes,” and “Type 1 diabetes prevention trials.”
Search yield	A total of 247 articles were retrieved across databases during the initial search; additional targeted references were incorporated during revision to address ancestry, biomarker strength, and prevention comments.
Inclusion rationale	Relevant human longitudinal cohorts, genetic studies, biomarker investigations, prevention and screening literature, consensus statements, and select mechanistic or animal studies were incorporated concerning preautoantibody biology, and no year restriction was applied because Stage 0 T1D is an emerging construct with limited specialized literature.
Exclusion rationale	Studies that only addressed established Stage 3 management and had no bearing on early risk, had no bearing on the pathophysiology of T1D, or were not available in English were eliminated.
Article screening process	Articles were distributed among authors, with each reviewer independently evaluating assigned sets of approximately 25 publications. Abstracts were screened for relevance.
Screening tool	A shared Excel spreadsheet was used to categorize articles as “yes,” “no,” or “maybe” for inclusion.
Screening outcomes	Included (“yes”): 96 articles; excluded (“no”): 65 articles; uncertain (“maybe”): 65 articles; unclassified: 20 articles.
Quality assessment	Methodological quality was assessed using the SANRA scale (Scale for the Assessment of Narrative Review Articles). After revision, the manuscript met five of six SANRA domains, corresponding to a provisional score of 10/12; limitations remain due to narrative design and incomplete quantitative synthesis.
Ethical considerations	As a narrative review of published literature, Institutional Review Board (IRB) approval, IACUC approval, and informed consent were not required.
Bias considerations	Because this is a narrative review, selection bias, publication bias, and author judgment in study selection are possible. These limitations are explicitly addressed in the limitations section.

The methodological quality of this narrative review was assessed using the SANRA framework, with a score of 10/12; remaining limitations include the nonsystematic nature of narrative synthesis and the limited ability to quantify effect sizes across heterogeneous biomarker studies. Because this article reviews only published literature, ethics committee approval, IACUC approval, and informed consent were not required. The broad inclusion strategy was chosen because Stage 0 T1D remains an emerging construct.

## 3. Background: The Eisenbarth Model Revisited With “Stage 0” Emphasis

Stage 0 is presented here as a proposed, hypothesis‐driven research framework that involves subtle immunological, metabolic, genetic, microbial, and environmental associations preceding islet autoantibody seroconversion. Genetic factors, especially HLA and nonHLA variants, may interact with viral exposures, changes in the gut microbiome, and early immune development. In this context, the conventional Eisenbarth model is being extended as a research construct to incorporate dynamic, multidimensional risk markers that may better reflect the complexity of early T1D development [17]. Section 4.6 elaborates on a provisional operational definition of Stage 0, emphasizing the absence of islet autoantibodies, normoglycemia, ancestry‐adjusted genetic risk, and candidate supportive biomarkers rather than a formal clinical diagnosis.

In Stage 1, individuals are asymptomatic but test positive for at least two islet autoantibodies (GAD, IA‐2, ZnT8), indicating early beta‐cell autoimmunity, yet still maintain normal glucose tolerance [4]. Genetic susceptibility plays a significant role, with high‐risk HLA Class 2 haplotypes, such as DR3‐DQ2 or DR4‐DQ8, influencing which autoantibodies appear first [18].

Stage 2 marks the start of dysglycemia while individuals remain asymptomatic. Metabolic dysfunction becomes quantifiable, as evidenced by diminished first‐phase insulin secretion and elevated proinsulin‐to‐insulin ratios, indicating beta‐cell stress and compromised insulin processing. People with very high ratios are more likely to progress to Stage 3 and may be considered for approved or trial‐based immune interventions, including teplizumab, where clinically indicated [19].

Stage 3 marks diabetes′s clinical onset, with autoimmune destruction causing insufficient insulin secretion and resulting in hyperglycemia. Symptoms include polyuria, polydipsia, weight loss, fatigue, and sometimes diabetic ketoacidosis, necessitating insulin therapy [4].

Diving T1D into established stages and early biological signals before autoimmunity (the proposed Stage 0 framework), as illustrated in Figure 1 [5, 6] and supported by the JDRF, the ES, and the ADA, can improve risk stratification and help design trials aimed at preserving *β*‐cell before irreversible damage occurs [4]. To understand how Stage 0 might eventually be operationalized for research, emerging evidence from genetics, immunology, metabolism, and environmental exposure must be integrated cautiously.

## 4. Emerging Evidence Relevant to a Proposed Stage 0

T1D is strongly related to HLA Class II on Chromosome 6. Recent studies identify primary haplotypes, including HLA‐DR4‐DQ8 and HLA‐DR3‐DQ2, that are associated with increased islet autoantibody positivity. Individuals with both haplotypes have a 35.0% chance of positive autoantibodies by Age 6; those with only HLA‐DR3‐DQ2 have a 19.9% chance, whereas those with only HLA‐DR4‐DQ8 have a 26.9% chance [5]. These genetic associations identified before clinical T1D warrant prioritization in proposed Stage 0 research, but they should not be treated as sufficient to define the disease.

### 4.1. Polygenic Risks in the Proposed “Stage 0” Framework Before T1D Development

The development of comprehensive genetic risk scores (GRSs) has allowed the integration of multiple diabetes‐associated loci, including the highly influential HLA regions, into a single quantitative measure of T1D susceptibility. The second‐generation GRS (GRS2) is a prominent example, incorporating 67 single‐nucleotide polymorphisms (SNPs): 14 within the HLA‐DR/DQ region, 21 across other HLA loci, and 32 in nonHLA genes. By combining these variants into a single score, GRS2 achieves greater sensitivity and specificity for predicting T1D risk than evaluating HLA and nonHLA variants, separately [20]. We ranked common HLA and nonHLA genetic variants linked to T1D by their ability to predict susceptibility in Table 2. Column 1 shows major HLA haplotypes most associated with T1D, whereas Column 2 includes sensitive nonHLA loci. These variants significantly influence an individual′s GRS for T1D, impacting disease prediction and risk stratification.

**Table 2:** Rank of common genetic variants in the early development of T1D.

Rank	HLA gene/haplotypes (Column 1)	NonHLA gene mutations (Column 2)
1	DR3‐DQ2.5 (DRB1*03:01*–*DQA1*05:01–DQB1∗02:01)	CTLA4 (cytotoxic T lymphocyte‐associated protein 4)
2	DR4‐DQ8.1 (DRB1*04:XX*–*DQA103:01*–*DQB1* ∗ *03:02*)	PTPN22 (protein‐tyrosine phosphatase nonreceptor type 22)
3	DR9‐DQ9.3 (DRB1*09:01*–*DQA103:02*–*DQB1* ∗ *03:03*)	IFIH1 (interferon‐induced helicase C domain 1; MDA‐5)
4	DR15‐DQ6.1 (DRB1*15:02*–*DQA101:03*–*DQB106:01*) and *DR15-DQ6.2* (*DRB1*15:01–DQA1*01:02*–*DQB1*06:02)	IL2RA (interleukin‐2 receptor alpha; CD25)
5	DR15‐DQ6.3 (DRB1*15:XX*–*DQA101:03*–*DQB106:03*) and *DR15-DQ6.9* (*DRB1*15:XX–DQA1*01:02*–*DQB1*06:09)	—

### 4.2. Ancestry Diversity and Global Applicability of Genetic Risk Models

Because most historical T1D GRSs were developed from cohorts of European ancestry, their use as a Stage 0 screening instrument should be approached with caution in nonEuropean populations. Michalek et al. established HLA‐ancestry datasets and demonstrated that HLA‐region effect sizes and model efficacy varied among ancestry groups. [21]. These findings support the use of ancestry‐adjusted or multiancestry models rather than the direct transfer of a European‐only threshold to global screening programs.

Studies in African‐ancestry populations similarly demonstrate that ancestry‐specific risk models can improve prediction. Onengut‐Gumuscu et al. reported that an African‐specific GRS achieved strong discrimination and outperformed a European‐derived score in African‐ancestry cohorts [22]. In population‐level analyses, applying European‐derived Type 1 Diabetes Genetic Risk Score (T1DGRS) thresholds substantially underidentified individuals of African and South Asian ancestry as high risk, underscoring the need for ancestry‐calibrated thresholds and validation in diverse cohorts for Stage 0 risk stratification [23].

Evidence from Asian cohorts also supports population‐specific HLA architecture. Chinese and Han Chinese studies have identified risk patterns and HLA haplotypes, including DR9‐related haplotypes and East Asian‐specific susceptibility haplotypes, that are not fully captured by European‐derived GRS models [24, 25]. Therefore, a globally applicable Stage 0 framework should define genetic risk using validated ancestry‐adjusted HLA/GRS tools and should avoid treating DR3‐DQ2 and DR4‐DQ8 as universally sufficient markers of susceptibility. Table 2 lists the most common HLA haplotypes that warrant attention to achieve a universal understanding of risk and support more equitable diagnosis and risk stratification.

### 4.3. Environmental Exposures, Viral Exposure, and Gut Dysbiosis in the Putative Stage 0 of T1D Progression

Environmental exposures may contribute to the Stage 0 interval preceding the onset of islet autoimmunity. Key exposures implicated in disease initiation include early viral infections, alterations in gut microbiome composition, and early‐life nutritional factors [26]. These factors may interact with high‐risk HLA genotypes to activate innate and adaptive immune pathways before *β*‐cell autoimmunity becomes detectable. Only a subset of genetically susceptible children develops autoantibody positivity, usually after infectious or inflammatory events. Thus, environmental factors are best described as risk modifiers associated with a genetically susceptible host rather than as deterministic causes [27].

Viral exposures, particularly enteroviruses, have been associated with loss of immune tolerance through activation of autoreactive CD8+ T lymphocytes, *β*‐cell injury, release of autoantigens, and the subsequent autoantibody formation [15, 28, 29]. Enteroviruses such as Coxsackievirus B1 (CVB1) can replicate in *β*‐cells, leading to inflammation and immune activation [30–32]. CVB1 exposure is associated with IAA but not with GAD65 autoantibodies, indicating that different viruses may be linked to distinct autoimmune responses [33]. These early‐life viral exposures, especially enteroviruses, are plausible risk modifiers rather than stand‐alone causes of progression from the putative Stage 0 interval toward islet autoimmunity.

**Figure 2:**
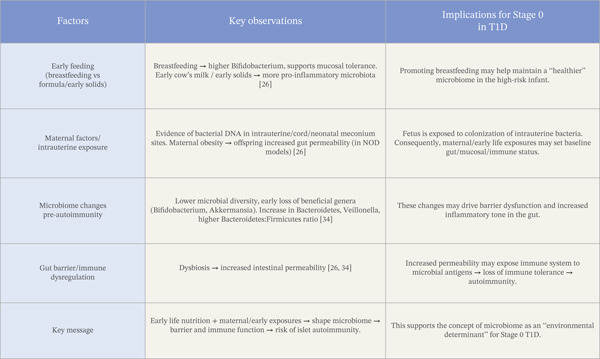
Environmental exposures, microbiome disruption, and proposed Stage 0 T1D risk pathways. Abbreviation: NOD, Nonobese diabetic.

Gut microbiome composition and early microbial exposures influence susceptibility to T1D during the putative Stage 0 interval. Infants who later progress to islet autoimmunity show reduced microbial diversity, early loss of beneficial genera such as *Bifidobacterium* and *Akkermansia*, and an increase in proinflammatory taxa, such as Bacteroidetes and *Veillonella*, long before seroconversion [34]. Breastfeeding promotes a bifidobacteria‐rich community that supports mucosal tolerance, whereas early cow′s milk exposure or early introduction of solid foods is associated with a more proinflammatory microbiota profile [26]. Molecular studies showing bacterial DNA in intrauterine and neonatal samples suggest that the microbial exposure may begin before birth. Nonobese diabetic (NOD) offspring from obese mothers had increased gut permeability and reduced villi/crypt ratios in the ileum, indicating enhanced permeability [26]. Figure 2 summarizes key environmental exposures, their effects on the microbiome, and their possible implications for the proposed Stage 0 framework.

**Figure 3:**
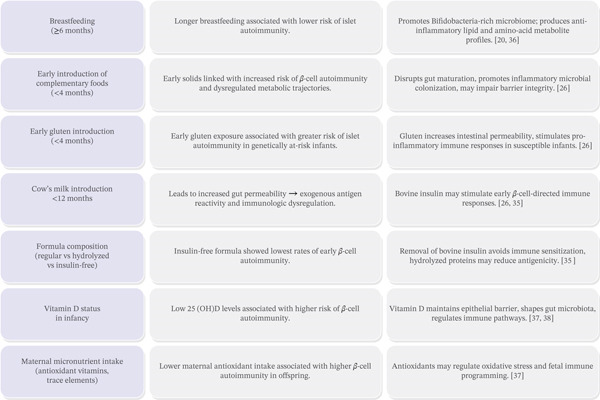
Early nutrition, immune‐metabolic signaling, and preautoimmune T1D risk.

Early‐life nutrition has been associated with risk of islet autoimmunity in genetically susceptible infants, but findings are not uniform across cohorts. Longer breastfeeding (> 6 months) is associated with better metabolic and microbial profiles, whereas early introduction of foods (gluten < 4 months) or cow′s milk (< 12 months) may increase risk [26]. The FINDIA trial suggested that infants on insulin‐free formula had lower *β*‐cell autoimmunity by Age 3, suggesting that bovine insulin may contribute to autoimmunity [35]. Metabolomic data from MIDIA indicate that feeding patterns shape lipid and amino acid profiles before seroconversion. Longer breastfeeding is associated with higher levels of phosphatidylcholines, sphingomyelins, lysophosphatidylcholines, and acylcarnitines, indicating reduced inflammation and improved membrane stability [36]. TEDDY data suggested that low 25 (OH) D levels in infancy are associated with increased risk of autoimmunity, likely by affecting epithelial integrity and microbiota–immune signaling [37, 38]. Figure 3 illustrates how early nutrition impacts microbial development and immune pathways during the putative Stage 0 interval.

The environmental literature is diverse. Associations with enterovirus, breastfeeding duration, cow′s milk exposure, gluten introduction timing, vitamin D levels, antibiotic use, mode of delivery, and microbiome composition are biologically plausible; however, effect sizes differ among cohorts and may be affected by geography, diet, sequencing techniques, age at sampling, HLA background, and family history. Human longitudinal cohorts offer the most robust translational evidence, whereas NOD mice and other animal studies are valuable for formulating mechanistic hypotheses but should not be regarded as direct clinical validation. This review categorizes these factors as associated or contributory exposures rather than definitive causal triggers of Stage 0 progression.

### 4.4. Metabolic and *β*‐Cell Stress: Endoplasmic Reticulum (ER) Stress, Oxidative Damage, and Insulin Secretory Dysfunction

Evidence suggests that, even before autoantibodies develop, *β*‐cells in genetically at‐risk children may experience stress during the putative Stage 0 interval. This stress involves mitochondrial and oxidative damage caused by altered lipid and acylcarnitine pathways [39]. Factors such as nutrient excess, imbalance, or maternal obesity can increase gut permeability and inflammation, thereby overwhelming *β*‐cell mitochondria and the ER in offspring. Such overload results in ER stress, oxidative harm, and reduced insulin secretion, making *β*‐cells more vulnerable and potentially predisposing genetically at‐risk children to diabetes [26]. Stressed *β*‐cells activate ER stress pathways, increase HLA Class I expression, and produce modified proteins that generate neoepitopes more easily detected by the immune system [26]. Overall, these metabolic and cellular stresses do not directly cause autoimmunity but may lower the threshold for environmental exposures, such as viral infections or gut inflammation, to initiate an autoimmune response [27].

### 4.5. Immune Dysregulation Before Autoimmunity: T cells, Cytokines, and Treg Changes in the Putative Preautoimmune Phase

Before autoimmune markers appear, the immune system maintains a balance between effector cells that control infection and regulatory T cells that preserve tolerance. In the putative preautoimmune phase, T cells may shift toward memory or effector subsets, autoreactive clones may expand, whereas cytokine profiles may become proinflammatory, thereby destabilizing immune equilibrium [34, 40]. Cytokines such as interferon alpha (IFN*α*), interferon gamma (IFN*γ*), interleukin 1 beta (IL‐1*β*), and tumor necrosis factor alpha (TNF*α*) may be elevated, reflecting early activation and inflammation [41]. Regulatory T cells may also exhibit altered suppressive capacity [34, 40]. These changes may precede seroconversion and loss of tolerance; in T1D, *β*‐cell injury is thought to be driven primarily by autoreactive T cells, whereas islet autoantibodies mark the transition to established autoimmunity. AntiCD3 therapy in Stage 2 T1D illustrates that modifying T cell responses can delay disease progression [42]. Thus, understanding early changes in T cells, cytokines, and regulatory T cells is critical for identifying candidate biomarkers and designing prevention trials. The proposed Stage 0 construct, therefore, has implications for screening, prevention, and public health policy but remains investigational.

### 4.6. Evidence Strength and Provisional Operational Definition of the Proposed Stage 0 Framework

The existing evidence does not currently support the recognition of Stage 0 as a recognized clinical stage. Stage 0 is optimally regarded as a provisional, research‐focused construct characterized by heightened genetic predisposition, the absence of islet autoantibodies, normoglycemia, and consistent early biological disturbances [4, 17, 20–25, 28, 43], and Table 3 ranks candidate Stage 0 markers by current evidence strength and translational readiness.

**Table 3:** Candidate Stage 0 biomarkers ranked by evidence strength and translational readiness.

Marker domain	Candidate markers	Current evidence strength	Provisional role in Stage 0 framework
Genetic susceptibility	Ancestry‐adjusted HLA haplotypes and validated T1D GRS/GRS2 or ancestry‐specific GRS [20–25]	Strongest and most reproducible; requires ancestry calibration [20–25]	Entry‐level risk stratification before autoantibody development [20–25, 43]
Islet autoantibodies	IAA, GAD65, IA‐2, ZnT8 [3, 4, 43–45]	Established for Stages 1–3, but absent by definition in Stage 0 [4, 43]	Used to confirm transition out of Stage 0 rather than to define Stage 0 [4, 43]
*β*‐cell stress/metabolic dysfunction	Proinsulin:C‐peptide ratio, altered lipidomics, amino acid and acylcarnitine profiles, and oxidative stress markers [11, 12, 19, 39, 46, 47]	Moderate to exploratory; findings require harmonized assays and prospective replication [11, 12, 39, 46, 47]	Supportive evidence of early *β*‐cell vulnerability when combined with high genetic risk [19, 39, 47]
Immune activation	Interferon‐related signatures, inflammatory cytokines, T cell phenotype shifts, and Treg functional changes [11, 40, 41, 47, 48]	Exploratory to moderate; variable across studies and methods [40, 41, 47, 48]	Mechanistic enrichment marker for research cohorts and early‐intervention trials [28, 40, 41, 47]
Microbiome/nutrition/ environment	Reduced microbial diversity, butyrate‐producing taxa changes, enterovirus exposure, vitamin D/nutrition patterns [13, 14, 26, 30, 33, 35–37, 39]	Exploratory; heterogeneous and context‐dependent [13, 26, 35–37, 39, 47]	Hypothesis‐generating modifiers of risk rather than stand‐alone diagnostic markers [26, 30, 33, 47]

A practical research definition could require: (1) absence of islet autoantibodies on confirmatory testing and normoglycemia [4, 43]; (2) high genetic risk based on an ancestry‐adjusted GRS or HLA model [20–25]; and (3) at least one replicated supportive signal, such as *β*‐cell stress, immune activation, or a reproducible metabolic/microbiome signature [11–13, 19, 26, 39–41, 47]. This definition should be used only for research enrichment until prospective studies establish validated thresholds, predictive values, and clinical utility.

## 5. Clinical, Scientific, and Public Health Implications of the Stage 0 Model

Screening may reduce the risk of DKA at diagnosis and can identify individuals eligible for education, surveillance, and approved therapy in Stage 2 [28, 43–45, 49]. Current practical screening tools include islet autoantibodies, genetic risk assessment, and metabolic monitoring; however, screening for the proposed Stage 0 framework would require ancestry‐adjusted genetic thresholds and validated supportive biomarkers before clinical implementation. Therefore, the strongest near‐term use of the Stage 0 framework is research enrichment rather than routine diagnosis.

### 5.1. Prevention and Early‐Intervention Strategies

Teplizumab provides the clearest proof of principle that immune intervention can delay the onset of clinical T1D after autoimmunity has been established. In the TN‐10 trial, teplizumab delayed progression from Stage 2 to Stage 3 T1D, and the current FDA label indicates that teplizumab‐mzwv delays Stage 3 T1D in adults and pediatric patients aged 1 year and older with Stage 2 T1D [42, 50, 51]. This evidence supports prevention‐oriented staging, but it does not validate treatment of autoantibody‐negative individuals; use in proposed Stage 0 cohorts remains limited to carefully monitored research until efficacy and safety are established.

Oral insulin trials illustrate both the promise and the challenges of antigen‐specific prevention. In TrialNet, oral insulin did not significantly prevent diabetes in the overall autoantibody‐positive cohort, although earlier DPT‐1 analyses suggested potential signals in subgroups with higher insulin autoantibody titers or greater metabolic risk [52, 53].

Abatacept, a CTLA4‐Ig costimulation modulator, has also been tested for delaying progression in at‐risk relatives. In a randomized TrialNet study of Stage 1 relatives, abatacept affected immune cell subsets and preserved aspects of insulin secretion during treatment, but it did not significantly delay progression to glucose intolerance or clinical T1D [54]. Abatacept, therefore, remains investigational for prevention and may be more informative for mechanistic enrichment or combination strategies than as a stand‐alone Stage 0 intervention.

Antigen‐specific tolerance strategies, including oral or intranasal insulin, GAD‐alum, peptide immunotherapies, DNA‐based approaches, and tolerogenic dendritic cell platforms, aim to restore immune tolerance without broad immunosuppression [29]. Their mixed results suggest that timing, dose, antigen selection, HLA background, immune endotype, and combination with immune‐modulating agents may determine efficacy. The proposed Stage 0 period could be attractive for these strategies because broad autoimmunity has not yet emerged, but trials must avoid overstating risk or treating children without a validated likelihood of progression.

Microbiome‐ and nutrition‐targeted interventions are conceptually relevant because early feeding patterns, vitamin D status, enteroviral exposure, antibiotic use, gut barrier function, and microbial diversity may influence mucosal tolerance [26, 34–38]. Potential approaches include breastfeeding support, correction of vitamin D deficiency when present, optimization of dietary patterns, probiotics or prebiotics, fecal microbiota‐based strategies, and enterovirus‐targeted vaccination. At present, these approaches should be framed as risk‐modifying or hypothesis‐generating rather than proven T1D prevention in autoantibody‐negative children.

A practical screening‐prevention pathway would be tiered: First, identify genetic or family‐history risk with ancestry‐calibrated tools; then perform confirmatory islet autoantibody screening; then use OGTT, HbA1C, C‐peptide, and other metabolic monitoring to distinguish Stage 1, Stage 2, and clinical diabetes; and finally link eligible individuals to counseling, structured follow‐up, teplizumab where indicated, or prevention trials [43–45, 55]. For proposed Stage 0 research, genetic risk plus replicated supportive biomarkers may enrich cohorts, but families should be counseled that Stage 0 is investigational and does not represent a diagnosis of T1D. Table 4 provides preventive and early intervention strategies that may be implemented to the proposed Stage 0 framework.

**Table 4:** Prevention and early‐intervention strategies relevant to the proposed Stage 0 framework.

Strategy	Current evidence and use	Implications for proposed Stage 0 research
Teplizumab/antiCD3 immune modulation	FDA‐approved to delay Stage 3 T1D in Stage 2 patients; TN‐10 showed delayed diagnosis [42, 50, 51].	Proof of principle for immune prevention after autoimmunity is established; not validated for autoantibody‐negative Stage 0 use.
Oral insulin and antigen‐specific insulin exposure	Overall TrialNet oral insulin results were not significant, but selected high‐risk subgroups showed possible metabolic or timing signals [52, 53].	Requires biomarker‐enriched trial design; not recommended for routine prevention outside clinical trials.
Abatacept/costimulation modulation	Stage 1 TrailNet study affected immune subsets and insulin secretion but did not significantly delay glucose intolerance or T1D [54].	May inform immune endotyping or combination approaches; remains investigational.
Antigen‐specific tolerance platforms	Oral/intranasal insulin, GAD‐alum, peptide, DNA, and tolerogenic dendritic cell approaches have mixed evidence [29].	Potentially attractive before broad immunity, but timing, HLA background, antigen choice, and safety require validation.
Microbiome, nutrition, vitamin D, and enterovirus‐targeted approaches	Associations exist for feeding, microbiome composition, vitamin D status, and enteroviral exposure, but casual evidence is heterogeneous [26, 34–38].	Should be framed as risk modification or hypothesis generation until prospective trials demonstrate prevention.
Tiered screening‐prevention pathway	Genetic/family‐history risk, autoantibody screening, metabolic staging, counseling, monitoring, and trial or approved‐therapy referral [43–45, 55].	Most feasible near‐term pathway: Stage 0 biomarker screening should remain research‐based until thresholds are validated.

Screening programs can also support prevention trials before symptom development. Ethical and communication factors are vital in the proposed Stage 0 models because informing families that a child has an elevated risk may cause anxiety, even when distress is generally temporary [56]. Supported education, confirmatory testing, clear risk communication, privacy safeguards, and structured follow‐up are essential to mitigate these concerns.

Implementation challenges include infrastructure, costs, long‐term follow‐up, ancestry calibration, psychological distress, and data privacy. Tiered approaches that begin with validated genetic or family‐history risk assessment and proceed to autoantibody and metabolic monitoring may optimize resource use [44, 45]. Education and counseling should assist at every stage of the program by clarifying risks, follow‐up steps, and available interventions. Table 5 summarizes the key features of the proposed Stage 0 and established Stages 1–3, emphasizing prevention potential.

**Table 5:** Key features of proposed Stage 0 vs. Stages 1–3 T1D.

Feature	Stage 0	Stage 1	Stage 2	Stage 3
Autoantibodies	Absent	Present	Present	Present
Glucose	Normal	Normal	Dysglycemia	Diabetes
Immune activation	Early	Established	Active	Advanced
Prevention potential	Highest	Moderate	Limited	None

## 6. Future Research Directions and Clinical Implications of Proposed Stage 0 Identification

**Figure 4:**
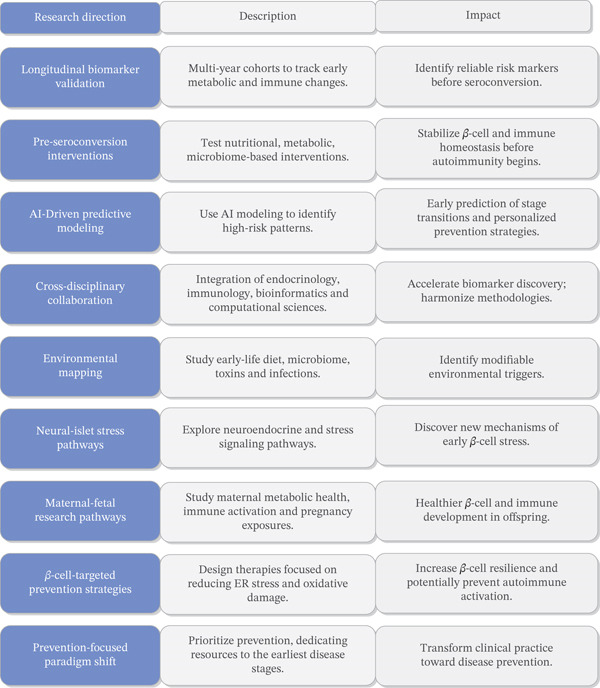
Research direction in Stage 0 T1D. Abbreviation: AI, artificial intelligence.

Future research should prospectively test whether a proposed Stage 0 framework improves prediction beyond established genetic and autoantibody screening. Key focus areas encompass multiancestry validation of HLA/GRS thresholds, standardized assays for *β*‐cell stress and immune activation, reproducible microbiome and metabolomic signatures, and trials assessing whether early intervention can prevent seroconversion, mitigate DKA at diagnosis, or preserve *β*‐cell function. Artificial intelligence (AI)‐based predictive models could be developed to forecast transitions between risk states, but such models will require transparent validation, bias assessment, and collaboration among endocrinologists, immunologists, geneticists, epidemiologists, and bioinformaticians. Focusing on proposed Stage 0 framework shifts the research goal from earlier detection alone toward evidence‐based prevention of T1D. Figure 4 outlines the main directions for future research.

Potential clinical implications of proposed stage 0 identification may:

• Enable early‐life risk stratification when validated biomarkers are available.
• Support population‐based screening strategies after ancestry‐calibrated validation.
• Guide immunomodulatory, antigen‐specific, metabolic, or microbiome‐targeted prevention trials.
• Reduce the risk of DKA at diagnosis through earlier education and monitoring.
• Support personalized prevention strategies if benefit, safety, and cost‐effectiveness are demonstrated.

## 7. Limitations

Several limitations should be considered when interpreting the Stage 0 framework. First, this review design is narrative rather than systematic, creating a risk of selection bias, publication bias, and incomplete capture of negative studies. Second, many studies are observational and enriched for relatives or genetically high‐risk children, which may overestimate predictive performance when applied to the general population. Third, effect sizes are not consistently reported across biomarker studies, and assay platforms differ for cytokines, metabolomics, microbial sequencing, and beta‐cell stress markers. Fourth, human cohort data and animal‐model findings address different questions: human longitudinal cohorts are more relevant for prediction, whereas animal models help test mechanisms but may not replicate human timing, genetics, or environmental exposures. These limitations support cautious, association‐based language and emphasize the need for prospective, multiancestry validation.

Preautoimmune “Stage 0” lacks biomarkers that define its distinctive features for diagnosis, and preliminary metabolic alterations, including changes in lipid and amino acid levels, have been reported but require further validation [6, 11, 12]. Microbiome findings are difficult to generalize across diets, geographies, antibiotic exposures, and sequencing platforms, making it challenging to generalize across different populations [5, 13, 26, 34]. *β*‐cell stress markers and subtle insulin‐secretory abnormalities may occur in conditions other than T1D and therefore lack disease specificity [19, 46]. Increases in inflammatory cytokines or early shifts in T cell populations are promising but exhibit substantial variability across studies [34, 40, 57]. Overall, there is still no reliable standard method for identifying Stage 0, reinforcing that it should remain a research construct.

Generalizability is also limited. Many genetic risk studies and polygenic risk scores were developed in European‐ancestry populations, reducing transferability to African, South Asian, and admixed populations [21–25, 58]. Environmental factors, such as viral exposure, nutrition, and microbial development, also vary across populations. These differences underscore the need for multiancestry prospective cohorts, standardized biomarker assays, careful consent, privacy safeguards for genetic data, and counseling that communicates uncertainty without implying clinical diagnosis [44, 45, 55].

## 8. Conclusion

Evidence supports the hypothesis that a proposed Stage 0 framework may capture biological risk factors prior to islet autoantibody formation, including genetic susceptibility, immune activation, metabolic stress, and environmental exposures. However, Stage 0 should currently be regarded as a research framework rather than an established clinical stage. Standardizing biomarkers, validating ancestry‐adjusted genetic models, and integrating genetic, metabolic, immune, and environmental profiling will be essential before this framework can be considered for routine clinical use. Future interventional trials should test whether individuals identified through this provisional framework benefit from prevention strategies that reduce seroconversion, DKA at diagnosis, or lifetime disease burden without causing unnecessary anxiety, overtreatment, or inequitable access.

## Author Contributions

**Fadi Ali Jamaleddin Ahmad:** conceptualization. **Juan José Zambrano Valenzuela:** methodology. **Maya Puhachova, Huda K. Klair, Balamurali Hariharan, Iffat Imtiaz, Juan José Zambrano Valenzuela, Essra Hassan Adam Alradi, Elizabeth Gbobbo, Faiqa Zahoor, Hassan Shah, Viraj Kale, Niketa Narasimhan, Fadi Ali Jamaleddin Ahmad:** literature search and screening, writing – original draft, writing – review and editing, and visualization. **Fadi Ali Jamaleddin Ahmad and Elizabeth Gbobbo:** supervision and project administration.

## Funding

No funding was received for this manuscript.

## Disclosure

All authors have read and approved the final version of the manuscript. Elizabeth Gbobbo, corresponding author/manuscript guarantor, had full access to all data in this study and takes full responsibility for the integrity and accuracy of the data analysis. Before resubmission, the authors reviewed the cited references for retraction or major correction status using Zotero. No cited reference was found to be retracted. If a correction is identified during the journal production process, the authors will evaluate whether it affects citation relevance or interpretation.

## Ethics Statement

This article constitutes a narrative review of extant literature. Approval from the institutional review board and IACUC and informed consent were unnecessary because no human participants, animals, identifiable private information, or new patient‐level data were used.

## Conflicts of Interest

The authors declare no conflicts of interest.

## Supporting information


**Supporting Information** Additional supporting information can be found online in the Supporting Information section.

## Data Availability

Data sharing not applicable to this article as no datasets were generated or analyzed during the current study.
