# Supplementary material for: Extending the Eisenbarth Model: Stage 0 as a Provisional Framework for Early Risk Stratification and Prevention in Type 1 Diabetes
Source: J Diabetes Res. 2026 Jun 26;2026:9970365. doi: 10.1155/jdr/9970365 (PMC13307194; doi:10.1155/jdr/9970365)
Supplement: Supplementary file 1 — Supporting Information Additional supporting information can be found online in the Supporting Information section. [file JDR-2026-9970365-s001.docx]

In order to get your submission publication ready, please review the editorial office requests below and make the corresponding updates:

Screening Notes:

1. Upon checking we found that the co-author mail id is mismatch from both system and manuscript. kindly update it.

**Authors Response**:

Updated. The manuscript author details were revised to align the co-author email information with the submission metadata; The corresponding author will also verify that the Wiley submission system metadata matches the revised manuscript before final upload.

2. Kindly update the affiliation as in the order mentioned below both in system and manuscript file : "(1) the name of the department, (2) the name of the institution, and (3) the city, state and country".

**Authors Response**:

Updated. The affiliations in the manuscript have been revised to follow the requested order: department/school/faculty, institution, city, state, and country, using the author information provided.

3. The Affiliation in non-English language. Hence, please translate the non-English Affiliation to English and upload an updated manuscript file (Doc format) under the supplementary section.

**Authors Response**:

Updated. Non-English institution names have been translated into English in the manuscript. For example, Università Cattolica del Sacro Cuore was updated to Catholic University of the Sacred Heart, and Universidad Nacional de Colombia was updated to National University of Colombia.

4. Please upload editable versions of the high resolution figures under Figure file section. None of the figures can be edited. We need to be able to edit lines, arrowheads, and fonts to match the journal’s style. Each figure should be a separate Word, Excel, PowerPoint or PDF file which can be edited. Please note that jpg, bmp, png, and tif files cannot be edited by default. An update is required regarding the figures/tables for this submission. Additional information is required before this submission can be considered further.

**Authors Response**:

Updated. Editable high-resolution figure files have been prepared as separate PowerPoint files for Figures 1-4. Each figure was recreated using editable PowerPoint shapes, text boxes, and lines, allowing the journal to edit lines, arrowheads, and fonts as needed. Note: As we revised the citations in the figures, we encountered issues, so the PowerPoint contains the most up-to-date, error-free citations. The figure citations in the Word document are out of order; please disregard Figures 2 and 3, as Figures 1 and 4 remain correct.
